# Insight into the dual function of lipid phosphate phosphatase PgpB involved in two essential cell-envelope metabolic pathways in *Escherichia coli*

**DOI:** 10.1038/s41598-020-70047-5

**Published:** 2020-08-06

**Authors:** Xudong Tian, Rodolphe Auger, Guillaume Manat, Frédéric Kerff, Dominique Mengin-Lecreulx, Thierry Touzé

**Affiliations:** 1 Université Paris-Saclay, CEA, CNRS, Institute for Integrative Biology of the Cell (I2BC), 91198 Gif-sur-Yvette, France; 2 Centre d’Ingénierie des Protéines, InBioS, Université de Liège, Liège, Belgium

**Keywords:** Biochemistry, Microbiology

## Abstract

Ubiquitous PAP2 lipid phosphatases are involved in a wide array of central physiological functions. PgpB from *Escherichia coli* constitutes the archetype of this subfamily of membrane proteins. It displays a dual function by catalyzing the biosynthesis of two essential lipids, the phosphatidylglycerol (PG) and the undecaprenyl phosphate (C_55_-P). C_55_-P constitutes a lipid carrier allowing the translocation of peptidoglycan subunits across the plasma membrane. PG and C_55_-P are synthesized in a redundant manner by PgpB and other PAP2 and/or unrelated membrane phosphatases. Here, we show that PgpB is the sole, among these multiple phosphatases, displaying this dual activity. The inactivation of PgpB does not confer any apparent growth defect, but its inactivation together with another PAP2 alters the cell envelope integrity increasing the susceptibility to small hydrophobic compounds. Evidence is also provided of an interplay between PAP2s and the peptidoglycan polymerase PBP1A. In contrast to PGP hydrolysis, which relies on a His/Asp/His catalytic triad of PgpB, the mechanism of C_55_-PP hydrolysis appeared as only requiring the His/Asp diad, which led us to hypothesize distinct processes. Moreover, thermal stability analyses highlighted a substantial structural change upon phosphate binding by PgpB, supporting an induced-fit model of action.

## Introduction

Undecaprenyl phosphate (C_55_-P) plays an essential role in the biogenesis of bacterial envelope polysaccharides such as the peptidoglycan^1^. It is used as a lipid carrier allowing the translocation of polymer subunits across the plasma membrane to the outer site, where the polymers are assembled^2^ (Supplementary Fig. S1). The synthesis of C_55_-P proceeds via the hydrolysis of its precursor, the undecaprenyl pyrophosphate (C_55_-PP), itself being de novo synthesized at the cytosolic side of the plasma membrane or released during subunits polymerization at the outer side (Supplementary Fig. S1)^1^. Four integral membrane enzymes catalyzing C_55_-PP hydrolysis have been identified in *Escherichia coli*: BacA*,* PgpB, YbjG and LpxT (formerly YeiU)*,* which belong to two unrelated protein families: BacA and Phosphatidic Acid Phosphatases of type 2 (PAP2)^3–5^. None of these enzymes is essential for growth, but the simultaneous knockout of *bacA*, *pgpB* and *ybjG* genes elicits a lethal phenotype due to a default of C_55_-P supply^4^. These enzymes have their active sites oriented towards the periplasm, suggesting they may rather be involved in C_55_-PP recycling^5–10^. The mechanism of translocation of C_55_-P back to the inner side of the membrane is yet unknown. The structure of BacA, which is reminiscent to that of transporters, raised the hypothesis that it may also catalyze the flip of C_55_-P^7,10^. The lack of a known cytoplasm-oriented C_55_-PP phosphatase raised also the question as to whether BacA and PAP2s are required for the dephosphorylation of de novo synthesized C_55_-PP (Supplementary Fig. S1).

PgpB is involved in another essential metabolic pathway, i.e. the synthesis of phosphatidylglycerol (PG) from its precursor phosphatidylglycerol phosphate (PGP)^11^. In a similar way as for C_55_-PP hydrolysis, there is a plurality of enzymes involved in PGP hydrolysis as two other non-PAP2 PGP phosphatases, PgpA and PgpC, exist^12^. PgpB possesses a broad substrate spectrum as shown by its capacity to hydrolyze phosphatidic acid, lysophosphatidic acid and diacylglycerol pyrophosphate in addition to C_55_-PP and PGP^6,13,14^. Thus, PgpB connects membrane glycerophospholipids and cell-wall polysaccharides biosynthesis. Interestingly, LpxT was also shown to establish an unexpected link between the biosynthesis of cell-wall polysaccharides and outer membrane lipopolysaccharides (LPS) by catalyzing the transfer of the β phosphate group of C_55_-PP to the lipid A moiety of LPS^5^.

**Figure 1:**
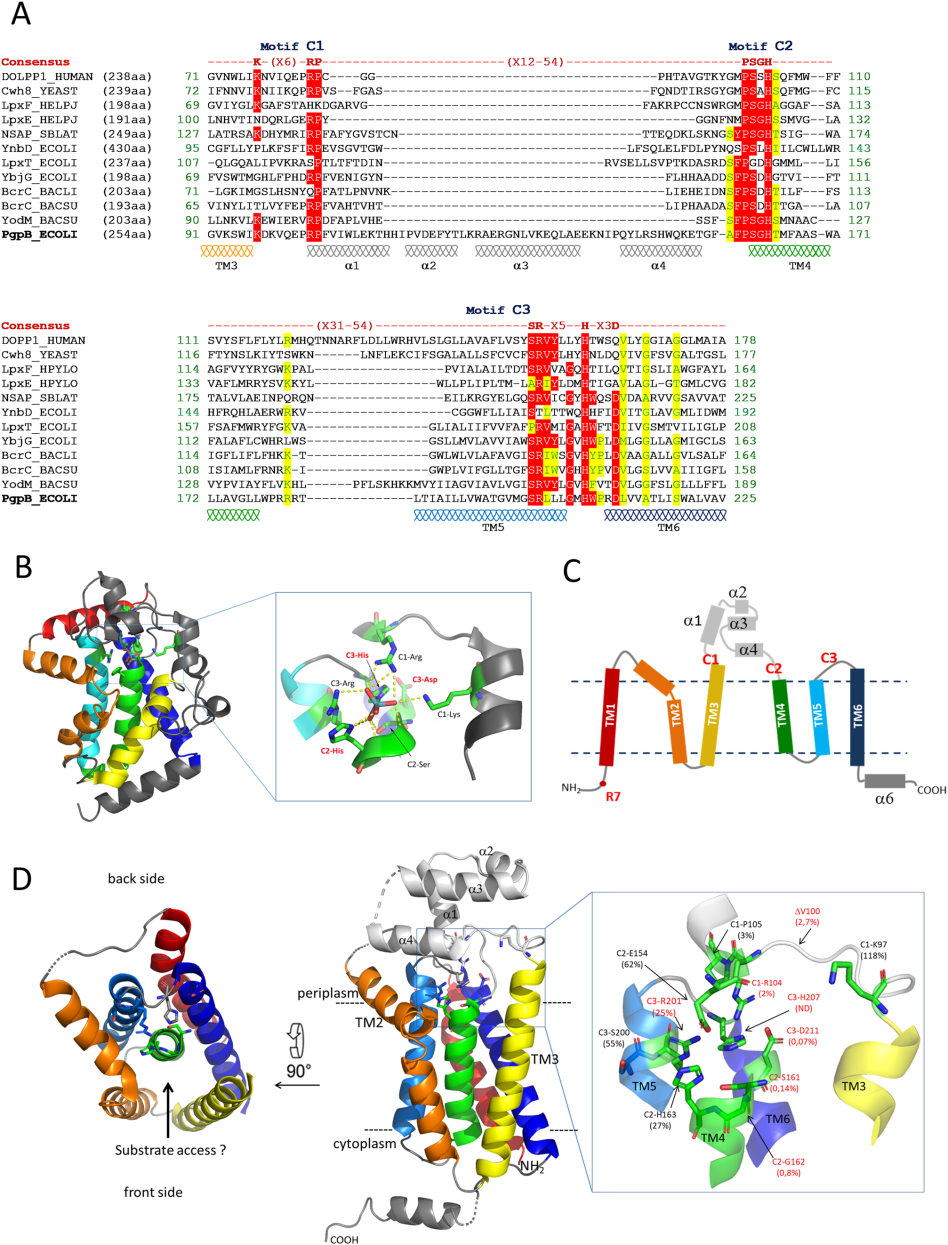
(**A**) Sequence alignment of PAP2 consensus regions of PgpB, YbjG, LpxT and YnbD from *E. coli*, YodM and BcrC from *B. subtilis* or *B. licheniformis,* dolichyl-pyrophosphate phosphatases from Human (DOLPP1) and *Saccharomyces cerevisiae* (Cwh8), lipid A phosphatases LpxE and LpxF from *H. pylori* and NSAP from *S. blattae*. Identical and similar residues are indicated on a red and a yellow background, respectively. (**B**) Cartoon representation of molybdate-bound NSAP structure (PDB 1EOI) with the core helix bundle represented in rainbow color gradient from the N-terminus in blue to the C-terminus in red. (**C**) Topology diagram of PgpB. (**D**) Cartoon representation of PgpB structure (PDB 4PX7). The signature residues are labeled in red or black depending on whether or not their mutation affects the protein activity in vivo, respectively, and the residual C_55_-PP phosphatase activity of each variant is shown in parentheses. The figure was generated with PyMol.

PAP2 proteins are defined by a signature sequence composed of three short motifs designated as C1, C2 and C3: K(X_6_)RP-(X_12-54_)-PSGH-(X_31-54_)-SR(X_5_)H(X_3_)D^15^ (Fig. 1A). The PAP2 family comprises soluble and membrane proteins^16^, raising the question as to whether they share similar mechanisms. The structures of soluble PAP2s were reported in their transition-state analog molybdate- and phosphate-bound forms^17,18^ (Fig. 1B). Their catalytic cycle is initiated by a nucleophilic attack of the substrate phosphoryl group by the histidine residue from motif C3 (C3-His), leading to the covalent binding of a phosphate group to a nitrogen atom of the imidazole ring^19,20^ (Fig. 1B). The formation of this phospho-enzyme intermediate is believed to be established via a charge-relay system involving the aspartate residue from motif C3 and the protonation of the substrate-leaving group by the histidine residue from motif C2. In a second step, the catalytic intermediate undergoes a nucleophilic attack by a water molecule, releasing inorganic phosphate. The C2-histidine is thought to act this time as a base to activate the water molecule.

The structures of PgpB and another membrane PAP2, YodM from *Bacillus subtilis,* were reported in their apo-form^13,21^, showing similar folding topologies as soluble PAP2s characterized by a core helix bundle (Fig. 1C,D). PgpB contains six transmembrane α-helices (TM), placing the signature residues at the membrane-periplasm interface (Fig. 1C,D). The C1 and C2 motifs of PgpB are separated by a 70-amino acid loop region, which shapes a small α-helical domain expanding into the periplasm (Fig. 1D).

In the present study, we showed that PgpB is able to sustain simultaneously PG and C_55_-P supplies in vivo, while the other lipid phosphatases are specific to their respective C_55_-PP or PGP substrates. Two previous studies provided contradictory results on the role of PgpB signature residues. Fan et al. reported that mutations of most signature residues completely abolished PgpB activity^13^. In contrast, Tong et al. reported that the triad H207/D211/H163 was essential for phosphatidic acid and lysophosphatidic acid hydrolysis, while H163 and D211 residues were dispensable for PGP hydrolysis^14^. In addition, the mutation of K97 by Fan et al. inactivated PgpB towards phosphatidic acid and lysophosphatidic acid, while the same mutation by Tong et al. increased the activity towards phosphatidic acid and PGP. Noteworthy, none of these studies assayed the C_55_-PP as substrate and no in vivo functional assays were supporting their findings. These observations prompted us to investigate further the role of invariant residues of PgpB with respect to its natural substrates. We then highlighted a discrepancy on the role of certain active site residues. In particular, R201 and H163 were found to be unnecessary for C_55_-PP hydrolysis, while they remained essential for PGP hydrolysis, which led us hypothesize two distinct mechanisms.

## Results

### PgpB displays an exclusive dual function

The *E. coli* BWTs*bacA* strain that is deleted of *bacA, pgpB* and *ybjG* genes and carries a temperature-sensitive plasmid with a copy of *bacA* (pMAK*bacA*), lyses after a shift from 30 to 42 °C due to a default in C_55_-P supply^4^. Given that LpxT also catalyzes the C_55_-PP dephosphorylation^5^, we generated here the BWTetra-Ts*bacA* strain deleted of all C_55_-PP phosphatases encoding genes and carrying pMAK*bacA* (Table 1). As expected, this strain displayed the same thermosensitivity as BWTs*bacA* (Table 2)*.* Likewise, the thermosensitive BWPGPTs strain, deleted of *pgpA, pgpB* and *pgpC* and carrying the rescuing pMAK*pgpB* plasmid had been generated^21^ (Tables 1, 2).

**Table 1:** Bacterial strains.

Strains	Genotype	Source
DH5α	*supE44, lacU169, hsdR17, recA1, endA1, gyr696, relA1, 80d lacZ*Δ*M15*	Invitrogen
C43(DE3)	*F-ompT, gal, hsdsB (r*_*BnB*_*), dcm, DE3*	Avidis
BW25113	*lacI*^q^*rrnB*_T14_ Δ*lacZ*_WJ16_*hsdR514* Δ*araBAD*_AH33_ Δ*rhaBAD*_LD78_	^31^
BWTs*bacA*	BW25113 Δ*bacA*, Δ*ybjG*, Δ*pgpB*::Kan^R^/pMAK*bacA*	^4^
BWTetra-Ts*bacA*	BW25113 Δ*bacA*, Δ*ybjG*, Δ*lpxT*, Δ*pgpB*::Kan^R^/pMAK*bacA*	This study
DMEG8	BW25113 Δ*bacA*, Δ*ybjG*, Δ*lpxT*	^4^
DMEG4	BW25113 Δ*pgpB*::Kan^R^	^4^
DMEG9	BW25113 Δ*ybjG*, Δ*lpxT*, Δ*pgpB*	^4^
BWPGPTs	BW25113 Δ*pgpA*, Δ*pgpC*, Δ*pgpB*::Cam^R^/pMAKkan*pgpB*	^21^
BW*pgpB*-single	BW25113 Δ*bacA*, Δ*ybjG*, Δ*lpxT*, Δ*pgpA*::Cam^R^, Δ*pgpC*::Kan^R^	This study
DMEG10	BW25113 Δ*bacA*, Δ*ybjG*, Δ*lpxT*, Δ*pgpA*::Cam^R^	^4^
JW2544	BW25113 Δ*pgpC*::Kan^R^	^30^
BWPAP2-less	BW25113 Δ*ybjG,* Δ*pgpB,* Δ*ynbD*::Kan^R^, Δ*lpxT*:Cam^R^	This study
DMEG12	BW25113 Δ*ybjG,* Δ*pgpB*::Kan^R^	^4^
BWΔ*ybjG*Δ*pgpB*	BW25113 Δ*ybjG*, Δ*pgpB*	This study
BWΔ*ynbD*::Kan^R^	BW25113 Δ*ynbD*::Kan^R^	This study
BWΔ*ybjG*Δ*pgpB*Δ*ynbD*	BW25113 Δ*ybjG*, Δ*pgpB,* Δ*ynbD*::Kan^R^	This study
DMEG3	BW25113 Δ*lpxT*:Cam^R^	^4^
BWΔ*bacA*	BW25113 Δ*bacA*	This study
BWΔ*lpxT*	BW25113 Δ*lpxT*	This study
BWΔ*pgpB*	BW25113 Δ*pgpB*	This study
BWΔ*ybjG*	BW25113 Δ*ybjG*	This study
DMEG1	BW25113 Δ*bacA*::Cam^R^	^4^
DMEG2	BW25113 Δ*ybjG*::Cam^R^	^4^
DMEG7	BW25113 Δ*ybjG*, Δ*lpxT*	^4^
BWΔ*lpxT*Δ*pgpB*	BW25113 Δ*lpxT*, Δ*pgpB*	This study
BWΔ*bacA*Δ*lpxT*Δ*pgpB*	BW25113 Δ*bacA*, Δ*lpxT*, Δ*pgpB*	This study
BW[PgpB][PBP1B]	BW25113 Δ*bacA*, Δ*ybjG*, Δ*lpxT*, Δ*mrcA*::Cam^R^	This study
BW[PgpB][PBP1A]	BW25113 Δ*bacA*, Δ*ybjG*, Δ*lpxT*, Δ*mrcB*::Cam^R^	This study
BW[YbjG][PBP1B]	BW25113 Δ*bacA*, Δ*pgpB*, Δ*lpxT*, Δ*mrcA*::Cam^R^	This study
BW[YbjG][PBP1A]	BW25113 Δ*bacA*, Δ*pgpB*, Δ*lpxT*, Δ*mrcB*::Cam^R^	This study
BW[BacA][PBP1B]	BW25113 Δ*ybjG*, Δ*pgpB*, Δ*lpxT*, Δ*mrcA*::Cam^R^	This study
BW[BacA][PBP1A]	BW25113 Δ*ybjG*, Δ*pgpB*, Δ*lpxT*, Δ*mrcB*::Cam^R^	This study
BWΔ*mrcA*::Cam^R^	BW25113 Δ*mrcA*::Cam^R^	^32^
BWΔ*mrcB*::Cam^R^	BW25113 Δ*mrcB*::Cam^R^	^32^

**Table 2:** In trans complementation of the thermosensitive strains.

Plasmid^a^	Protein	BWTs*bacA*		BWTetra-Ts*bacA*		BWPGPTs	
		− IPTG	+ IPTG^b^	− IPTG	+ IPTG	− IPTG	+ IPTG
None		−	−	−	−	−	−
p*Trc*His60	None	−	−	−	−	−	−
p*Trc*Bac30	BacA	+	NT	+	NT	−	−
p*Trc*H60*ybjG*	YbjG	+	NT	+	NT	−	−
p*Trc*H60*lpxT*	LpxT	−	−	−	−	−	−
p*Trc*H60*pgpB*	PgpB	+	+	+	+	+	+
p*Trc*H30*ynbD*	YnbD	−	−	−	−	−	−
p*Trc*H60*pgpA*	PgpA	−	−	−	−	+	+
p*Trc*H60*pgpC*	PgpC	−	−	−	−	+	+
**C1motif**							
pA727	K97A	+	NT	+	NT	+	
**pA730**	**ΔV100**	**−**	**+**	**−**	**+**	**+**	**NT**
**pA733**	**R104A**	**−**	**+**	**−**	**+**	**+**	**NT**
pA737	P105A	+	NT	+	NT	+	NT
**C2motif**							
pB877	E154A	+	NT	+	NT	+	NT
pA741	A158S	+	NT	+	NT	+	NT
pA745	F159A	+	NT	+	NT	+	NT
pA762	P160A	+	NT	+	NT	+	NT
**pA766**	**S161A**	**−**	**+**	**−**	**+**	**+**	**NT**
**pA771**	**G162A**	**+**	**+**	**−**	**+**	**+**	**NT**
**pA749**	**G162D**	**−**	**−**	**−**	**−**	**−**	**−**
pA798	H163A	+	NT	+	NT	+	NT
**pB881**	**E154A/H163A**	**+**	**+**	**−**	**+**	**−**	**+**
pA802	T164A	+	NT	+	NT	+	NT
**C3motif**							
pA807	S200A	+	NT	+	NT	+	NT
**pA812**	**R201A**	**−**	**+**	**−**	**+**	**+**	**NT**
pA817	L202A	+	NT	+	NT	+	NT
pA822	G205A	+	NT	+	NT	+	NT
**pA828**	**H207A**	**−**	**−**	**−**	**−**	**−**	**−**
pA833	W208A	+	NT	+	NT	+	NT
pA838	P209A	+	NT	+	NT	+	NT
**pA843**	**D211A**	**−**	**+**	**−**	**+**	**+**	**NT**
pA847	L212A	+	NT	+	NT	+	NT
pA852	A215G	+	NT	+	NT	+	NT
pA857	S219A	+	NT	+	NT	+	NT

^a^The plasmids carrying a copy of the different lipid phosphatase encoding genes or *pgpB* variants were tested for their ability to restore the growth at 42 °C of the three thermosensitive strains. + , normal growth at 42 °C; −, no growth at 42 °C; NT, not tested.

^b^The growth at 42 °C was also monitored in the presence of IPTG in the growth medium at 1 mM in all cases, except for *lpxT* overexpression (10 µM). In bold boxes are indicated the variants displaying a complete or partial defect of complementation.

We then tested the restoration of growth at 42 °C of the latter strains with p*Trc*-based plasmids carrying the different phosphatase-encoding genes under the control of a strong IPTG-inducible promoter (Supplementary Table S1). By a Blast search using the core region of PAP2 enzymes encompassing the consensus sequence, the *ynbD* gene was identified, which encodes a fourth putative membrane PAP2 in *E. coli* (Fig. 1A) whose function was yet to be established. Therefore, *ynbD* was also tested for its ability to complement the thermosensitive strains. An ectopic copy of *bacA*, *pgpB* and *ybjG* restored the growth at 42 °C of BWTs*bacA* and BWTetra-Ts*bacA*, with no requirement for IPTG. In contrast, *lpxT*, *ynbD*, *pgpA* and *pgpC* failed (Table 2). Of note, the overexpression of *lpxT* with 1 mM IPTG was toxic to all strains as judged from the lack of growth at both 30 °C and 42 °C. The expression was then carried out with 10 µM IPTG, which did not confer toxicity as judged from growth at 30 °C, but still did not complement. The *pgpA*, *pgpB* and *pgpC* genes complemented the BWPGPTs strain with no need for IPTG, while *bacA* and other PAP2 genes failed (Table 2). These data supported the evidence that PgpB is the sole among these lipid phosphatases being active on two such dissimilar substrates, i.e. a glycerophospholipid and a linear polyprenyl phosphate.

### PgpB can simultaneously supply C_55_-P and PG

To assess whether chromosomally expressed *pgpB* can simultaneously sustain C_55_-P and PG supplies, a strain disrupted of all PGP and C_55_-PP phosphatases encoding genes, except *pgpB,* was generated (Table 1)*.* This strain, called BW*pgpB*-single, grew similarly as the wild-type strain (WT) in standard growth conditions, supporting the promiscuous trait of PgpB in vivo and its ability to sustain both metabolic pathways simultaneously. To estimate the input of PgpB on C_55_-P and PG synthesis, both phosphatase activities present in the membranes of WT, BWΔ*pgpB* and BW*pgpB*-single cells were measured. The input of PgpB on C_55_-PP phosphatase activity was low as no significant decrease of this activity was observed upon *pgpB* knockout (Supplementary Table S2). Conversely, membranes from BW*pgpB*-single strain displayed only 7% of residual C_55_-PP phosphatase activity as compared to WT. In contrast, the input of PgpB on PGP phosphatase activity was high since a 93% decrease was observed upon *pgpB* deletion, while the activity was only weakened by *ca*. 27% in BW*pgpB*-single cells.

### Multiple PAP2 knockout mutants are sensitive to small hydrophobic compounds

**Figure 2:**
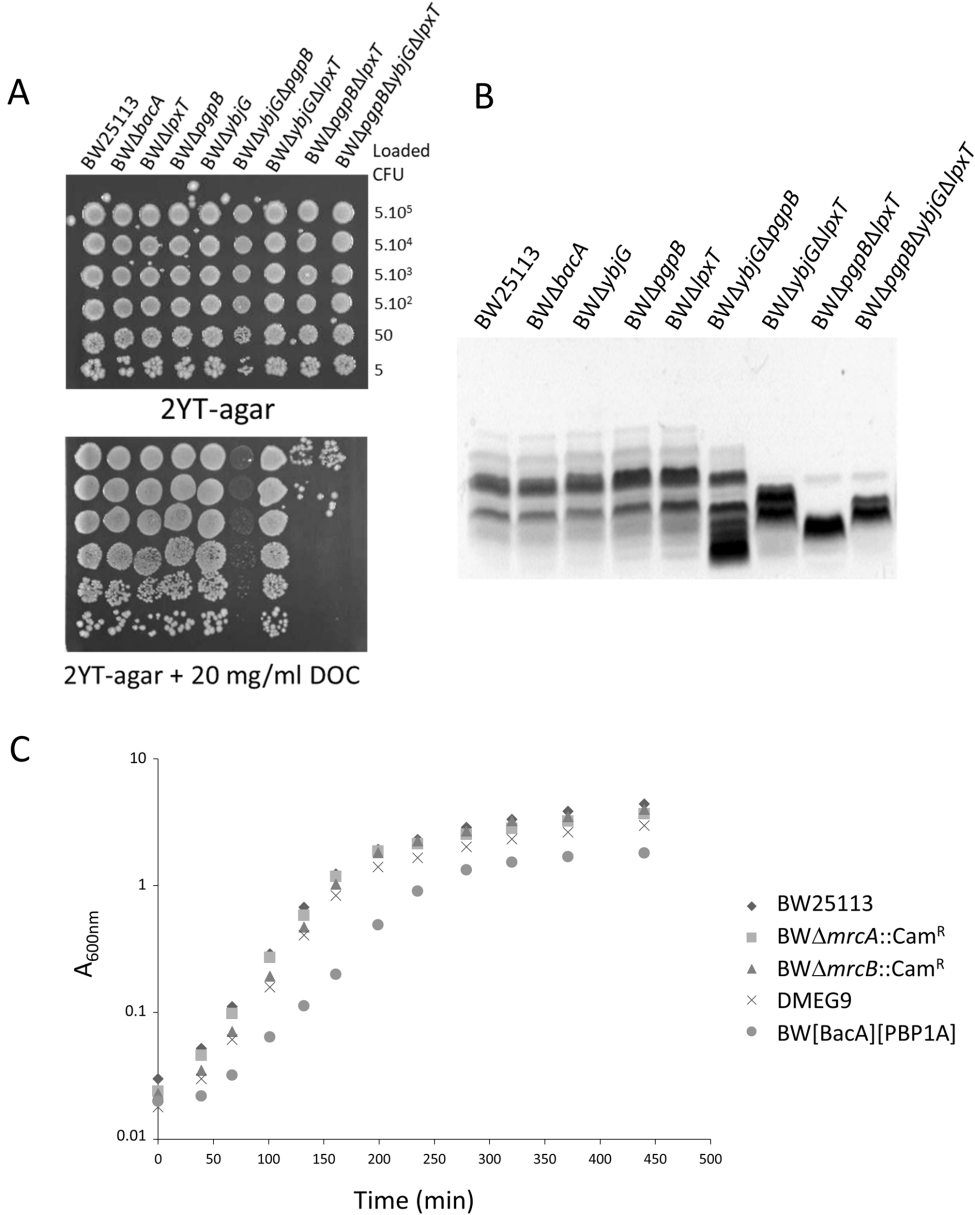
Phenotypic analyses of multiple PAP2-encoding genes knockout strains. (**A**) DOC sensitivity assay: 5 µl of serial dilutions of the indicated cells were laid down on 2YT-agar plates supplemented (down) or not (up) with 20 mg/ml of DOC. Approximate numbers of bacteria (CFU), according to the A_600nm_ of a liquid culture, which are present in the original drops, are indicated on the right. (**B**) Silver stained SDS–polyacrylamide gel showing migration patterns of LPS isolated from the different BW25113 WT and deletion mutant strains. (**C**) Fitness cost of simultaneous PAP2 and PBP inactivation. BW25113, BWΔ*mrcA*::Cam^R^ (PBP1A inactivated), BWΔ*mrcB*::Cam^R^ (PBP1B inactivated), DMEG9 (PgpB, YbjG and LpxT inactivated), BW[BacA][PBP1A] (PgpB, YbjG, LpxT and PBP1B inactivated) were grown in 2YT at 37 °C. Experiments were performed in triplicate, and the data shown are from independent experiments.

Except YnbD for which no function has yet been ascribed, all PAP2s are involved in membrane and/or cell wall biogenesis. We addressed whether the inactivation of all PAP2s was lethal. The corresponding strain, named BWPAP2-less, was generated (Table 1), showing the non-essentiality of PAP2 for growth in standard conditions. We further addressed the integrity of the cell envelope by monitoring the sensitivity of different PAP2 mutants to sodium deoxycholate (DOC), a small anionic detergent that targets the membranes. As shown in Fig. 2A, the growth of single deletion mutants and BWΔ*ybjG*Δ*lpxT* double-mutant was not altered as compared to WT on DOC-containing medium. In contrast, the BWΔ*ybjG*Δ*pgpB* mutant appeared translucent on DOC-containing medium, while the BWΔ*lpxT*Δ*pgpB* and BWΔ*lpxT*Δ*pgpB*Δ*ybjG* mutants exhibited a decrease of 4 log units in viable counts with respect to WT (Fig. 2A). The higher susceptibility to DOC of these strains was also observed in liquid culture medium and was characterized by an early arrest of growth, which could explain the translucent aspect of colonies on DOC-containing solid medium (Supplementary Fig. S2A). The BWΔ*lpxT*Δ*pgpB* and BWΔ*ybjG*Δ*pgpB* mutants also displayed higher susceptibility to Triton X100 as compared to WT, while only BWΔ*ybjG*Δ*pgpB* cells appeared more susceptible to sodium dodecyl sulfate (SDS) (Supplementary Fig. S2B). In contrast, none of these mutants showed an increase susceptibility to the cell wall-targeting antibiotic ampicillin (Supplementary Fig. S2B). The observed phenotype, i.e. an increased susceptibility to small hydrophobic compounds, thus rather suggests a defect in lipid bilayers, especially in the outer membrane, which is the permeability barrier to detergents and dyes. This feature being mainly ensured by the outermost LPS component, we therefore analyzed the migration pattern of LPS extracted from WT, single and double/triple deletion strains. These migration patterns clearly showed an altered profile in all PAP2 double and triple deletion strains, while single deletion strains displayed the same pattern as the WT (Fig. 2B). The *E. coli* K12 strains do not produce the O-antigen moiety, therefore their LPS are resumed to the lipid A–core region, whose normal heterogeneity is observed in WT and single deletion strains. The patterns of double/triple mutants show LPS species with apparent lower molecular weights and less heterogeneity, suggesting a default of LPS biosynthesis. Of note the patterns differ from one mutant to the other, which is correlated to their different tolerance to small hydrophobic compounds.

### Interplay between PAP2s and PBP1A

The C_55_-PP lipid is released at the outer side of the membrane by peptidoglycan glycosyltransferases (GTases) such as the penicillin-binding proteins (class A PBPs: PBP1A, PBP1B and PBP1C)^22^ or proteins from the SEDS family (shape, elongation, division, and sporulation, FtsW and RodA)^23^ (Supplementary Fig. S1). Thereafter, C_55_-PP is recycled by C_55_-PP phosphatases (Supplementary Fig. S1). Therefore, the C_55_-PP could be somehow channelled from peptidoglycan polymerases to a given phosphatase for an efficient recycling. The PBP1A and PBP1B play major roles in peptidoglycan polymerization and their simultaneous inactivation is lethal^24^. Recently, an interaction between PgpB and PBP1B was shown, highlighting a coupling event between peptidoglycan polymerization and C_55_-PP recycling^25^. We then addressed whether a strict interdependency exists between these major PBPs and C_55_-PP phosphatases. We inactivated PBP1A or PBP1B in mutants producing only one C_55_-PP phosphatase among BacA, PgpB and YbjG (Table 1). All possible combinations of knockout were obtained, showing that these PBPs can function with any C_55_-PP phosphatase and conversely. The fitness of these mutants was further examined by monitoring their growth in standard medium. As judged from Fig. 2C, the growth of the strain which only relies on PBP1A and BacA for peptidoglycan synthesis and C_55_-PP recycling, respectively, displayed a significant increase of its doubling time as compared to WT (39 min for BW[BacA][PBP1A] versus 27 min for WT). All the other mutants displayed a similar growth as WT. In conclusion, the inactivation of PAP2s has a significant impact on cell physiology as judged from DOC sensitivity and fitness change upon PBP1B inactivation.

### PgpB mutagenesis

To get insights into the dual function of PgpB, we investigated the role of PAP2 conserved residues on C_55_-P and PGP hydrolysis. Based on PAP2 sequences alignment and PgpB structure, 23 residues were selected for alanine mutagenesis. The A158 residue was substituted by Ser because Ser is present at this position in the other C_55_-PP phosphatases. The C2-G162 (glycine residue from motif C2) was also substituted by Asp, given the presence of Asp in the other C_55_-PP phosphatases (Fig. 1A). The TM3 is loosely packed to the rest of the protein generating a V-shaped cleft alongside the protein (Fig. 1D), where substrates were hypothesized to enter the catalytic site^13^. The top of TM3 is connected to the periplasmic domain via a 10-amino acid loop likely responsible for this loose packing and the positioning the C1-K97 signature residue away from the catalytic pocket (Fig. 1D). We addressed whether the length of this loop was important for PgpB activity by shortening it by one residue (ΔV100 variant). The mutagenesis was performed on the p*Trc*H60*pgpB* plasmid. All variants were tested for their activity by in vivo complementation assays and by kinetic analyses as described here after.

### Complementation of thermosensitive strains with PgpB variants

Out of 24 variants, eight failed to restore the growth of BWTetra-Ts*bacA* at 42 °C in the absence of IPTG: ΔV100, R104A from motif C1; S161A, G162A, G162D from motif C2, and R201A, H207A and D211A from motif C3 (Table 1). In the presence of IPTG, only G162D and H207A variants remained unable to complement. The same results were obtained with BWTs*bacA* strain (i.e. *lpxT* present), except with G162A variant, which complemented even without IPTG (Table 1). Upon disruption of the other C_55_-PP phosphatases, LpxT was unable to supply C_55_-P as judged from the thermosensitivity of BWTs*bacA* strain. Interestingly, we showed here that LpxT contributes to the growth at 42 °C of BWTs*bacA* producing the G162A PgpB variant since the same variant did not complement BWTetra-Ts*bacA*.

The thermosensitive BWPGPTs strain was complemented by all variants, except G162D and H207A, regardless the presence of IPTG (Table 1). To confirm that the lack of complementation was not due to an excessive protein instability or differential expression, the amount of variants present in native membranes was probed by immunoblotting. The data indicated that all variants were present at similar levels as the WT protein, except ΔV100 and G162A, which showed a slight decrease (about 20% less protein as judged from densitometry quantification) (Supplementary Fig. S3). We compared the level of expression of *pgpB* from the chromosomal copy *versus* the plasmid vector. The amount of *pgpB* transcript originating from p*Trc*H60*pgpB* plasmid was by 30- and 2000-fold higher as compared to the chromosomal expression, without and with IPTG, respectively (Supplementary Table S3).

### Kinetic analysis of PgpB variants

All PgpB variants were purified to homogeneity with similar yields as the WT protein, except C1-P105A for which a tenfold lower amount of protein was obtained. P105 is located at the N-terminus of the periplasmic domain, where it directs a tight turn of the protein backbone (Fig. 1D); its substitution may therefore alter the folding of this domain and the overall stability of the protein. The activity of PgpB variants towards C_55_-PP and 16:0 PGP were determined (Table 3). With the exception of C3-R201A (see below), a good correlation between in vivo functional complementation and in vitro enzymatic activity was observed (Tables 2, 3). Of note, a very low level of enzymatic activity appeared as sufficient for complementation, corresponding to 3% and 0.03% of residual activity for C_55_-PP and PGP hydrolysis, respectively (i.e. below this threshold, the complementation failed).

**Table 3:** Phosphatase activities of PgpB variants.

Protein	% of wt PgpB activity	
	C_55_-PP^a^	PGP^a^
PgpB	100 ± 16 (730 ± 120 nmol min^−1^ mg^−1^)	100 ± 19 (3,300 ± 630 nmol min^−1^ mg^−1^)
**C1motif**		
K97A	118 ± 12	298 ± 55
ΔV100	2.7 ± 0.6	0.4 ± 0.1
R104A	2.0 ± 0.4	0.4 ± 0.1
P105A	3 ± 1	0.4 ± 0.3
**C2motif**		
E154A	62 ± 11	61 ± 5
P160A	13 ± 2	2.5 ± 0.1
S161A	0.14 ± 0.08	0.03 ± 0.01
G162A	0.8 ± 0.1	0.2 ± 0.04
G162D	ND^b^	0.03 ± 0.02
H163A	27 ± 3	0.09 ± 0.03
E154A/H163A	10 ± 2	0.03 ± 0.01
**C3motif**		
S200A	55 ± 7	162 ± 23
R201A	25 ± 6	0.3 ± 0.1
H207A	ND	ND
W208A	18 ± 4	24 ± 2
D211A	0.07 ± 0.02	0.1 ± 0.1

^a^The enzymatic activity was measured in the presence of 50 µM of [^14^C]C_55_-PP or [^14^C]PGP substrate and an appropriate amount of enzyme to obtain less than 30% of hydrolysis. The product and substrate were separated by TLC and subsequently quantified by radioactivity counting.

^b^*ND* no detectable activity with up to 2 µg of pure protein.

### Identification of key residues for C_55_-PP and PGP hydrolysis

The C2-G162D and C3-H207A variants that were totally inactive in vivo displayed no or virtually no activity in vitro (Table 3). This is consistent with the catalytic role assigned to the histidine residue from motif C3 i.e. the nucleophilic attack of the P–O substrate bond, yielding the phosphohistidine intermediate. The G162D residue is positioned 6 Å below H207 in the catalytic pocket (Fig. 1D). A side chain will likely protrude toward the interior of the cleft, which is formed by TM3 translation and was hypothesized to be the substrate entrance. This may thus block substrate binding via steric hindrance and/or electrostatic repulsion of the phosphate, likely explaining a more severe defect created by the G162D rather than G162A mutation.

The C3-D211A variant displayed the lowest activity ever detected on C_55_-PP substrate and its activity on PGP was also severely decreased. For unknown reasons, our results are in marked contrast with those reported by Tong et al. according to the impact of D211A mutation on PGP hydrolysis as they described 50% of residual activity *versus* 0.1% in our study^14^. This C3-Asp residue was hypothesized to be involved in a charge relay with the C3-His residue (H207 in PgpB). In this model, the C3-Asp carboxylate establishes hydrogen bonding with ND1-H from C3-His, maintaining the NE2 atom unprotonated with the lone pair well positioned for the nucleophilic attack of the phosphoryl group. Consistent with this model, the carboxylate group from D211 lies within hydrogen bonding distance to the ND1 atom of H207 (Fig. 1D). It may also stabilize the positive charge displayed by H207 on the phospho-enzyme intermediate. Hence, our findings and the structure of PgpB support such a central role for C3-Asp in catalysis. Taking into account that D211 may keep the catalytic H207 residue in a favorable state for the nucleophilic attack, there is no reason why there should be a differential impact of D211 substitution according to the substrate such as observed by Tong et al.^14^.

The C2-S161A variant displayed an activity of the same order of magnitude as that determined for C3-D211A on both substrates. This C2-Ser residue adopts a conserved position within the catalytic pocket of PgpB as compared to soluble counterparts, where it was assumed to stabilize the bound phosphate group through hydrogen bonding of its hydroxyl side chain with a phosphate oxygen atom.

### Role of residues from motif C1

The C1-ΔV100 and C1-R104A variants displayed the same activities on both substrates, which were about ten-fold higher than that of D211A and S161A variants (Table 3). This suggested that shortening the loop connecting TM3 to the periplasmic domain might displace the C1-R104 residue away from its native position in such a way that this residue cannot exert its role anymore. Thus, shortening this loop does not seem to induce a translation of TM3 α-helix closer to the core helix. The side chain guanidinium group of R104 points toward the center of the catalytic pocket (Fig. 1D); it may thus ensure phosphate oxygen bonding and/or phosphoenzyme stabilization through electrostatic interaction.

The C1-K97A variant did not display any decrease in its hydrolytic activity as compared to the WT protein, PGP hydrolysis being even increased by threefold. Tong et al. also reported a 70% increase of PGP hydrolysis with this variant, while the hydrolysis of lyso substrates (lysophosphatidic acid and sphingosine-1-phosphate) was down to less than 10% of relative activity, suggesting that it might be involved in substrate selectivity regarding mono- versus diacyl substrates^14^. Nevertheless, here we found that this residue was also not involved in binding and/or catalysis of the single chain polyprenyl substrate.

### C3-R201A variant presents a paradox

**Figure 3:**
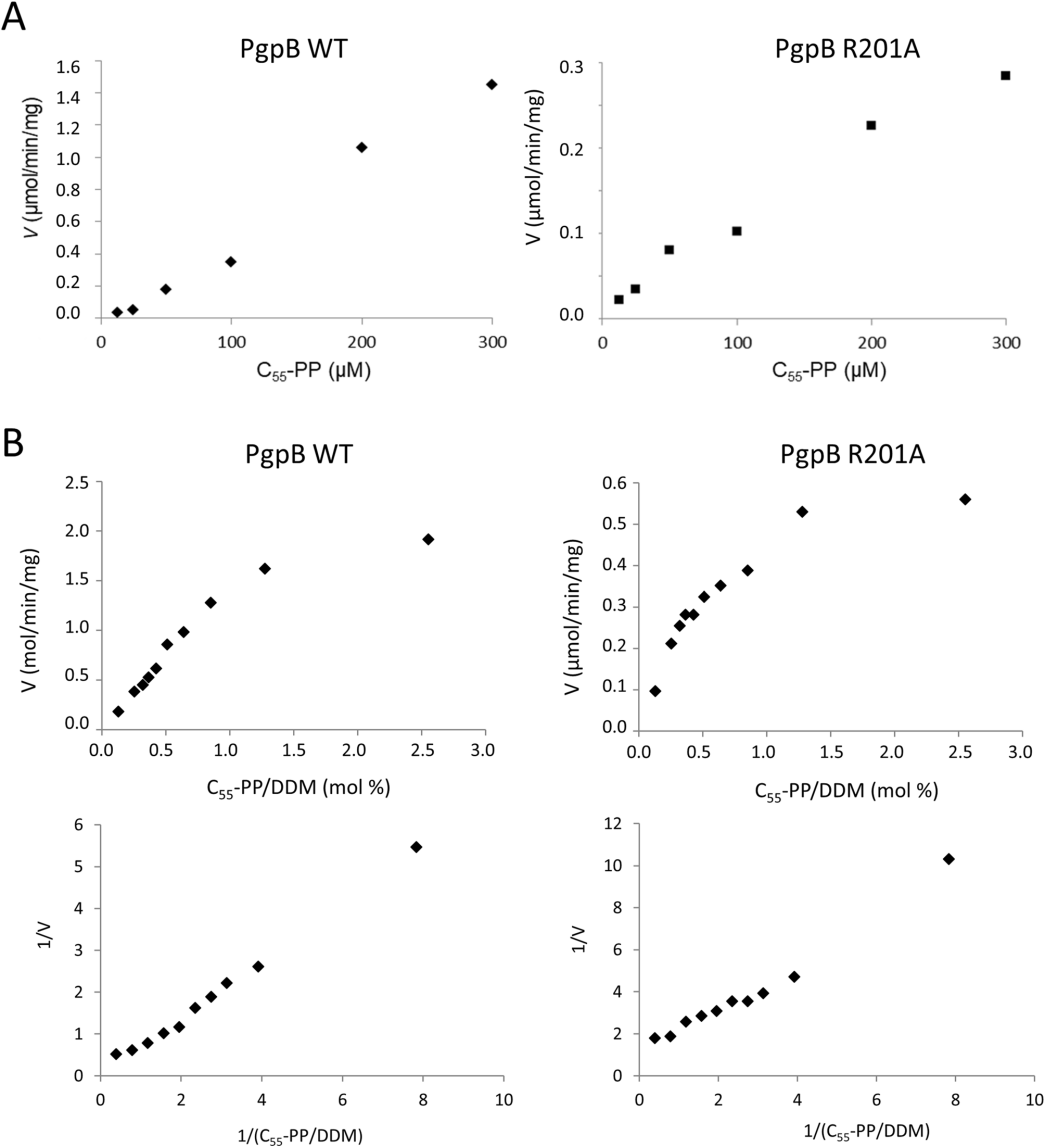
Kinetic studies of WT PgpB (left panels) and R201A variant (right panels). (**A**) The C_55_-PP phosphatase activity was measured as a function of the bulk concentration of substrate but no saturation phenomenon was observed in the range of concentrations that could be tested. (**B**) The C_55_-PP phosphatase activity was measured with a fixed concentration of substrate (50 µM) but various concentrations of DDM to reflect the dilution of [^14^C]C_55_-PP at the micellar surface, and the results are expressed as a function of the C_55_-PP/DDM mole ratio (mol%). The replot of 1/*v *versus the reciprocal of the C_55_-PP/DDM mole ratio is also represented.

Surprisingly, the C3-R201A variant displayed a relatively high activity on C_55_-PP (~ 25% of residual activity), while it was unable to complement thermosensitive strains altered in the C_55_-P pathway in absence of IPTG. This variant showed tenfold higher activity than R104A on C_55_-PP, while both variants displayed similar activities on PGP (~ 0.3% of residual activity). To investigate the reason why R201A variant was so particularly altered in vivo with respect to C_55_-P hydrolysis, we determined its kinetic parameters. The C_55_-PP phosphatase activity was measured as a function of the bulk concentration of substrate, but no parameters could be determined as no saturation was observed (Fig. 3A). We then measured the activity as a function of C_55_-PP/DDM detergent ratio (mol%) to reflect surface dilution kinetics (Fig. 3B). The velocity of hydrolysis was reduced by 75% with R201A variant as compared to WT at all ratio and the apparent *V*_max_ were 0.7 and 3.2 µmol/min/mg, respectively. The apparent *K*_*m*_ were found to be 1.4 and 0.6 mol% for WT and R201A variant, respectively. Thus, the apparent affinity could not explain the observed paradox.

### C_55_-PP and PGP hydrolysis are distinguishable

As already mentioned, the His residue from motif C2 (C2-H163) was hypothesized to be part of the catalytic triad (Fig. 1), being responsible for the protonation of the leaving group and the activation of a water molecule for nucleophilic attack of the phospho-enzyme intermediate. Surprisingly, the C2-H163A variant displayed 27% of residual activity on C_55_-PP, while its activity was down to 0.09% on PGP. These results were in contrast with those from Tong et al., who reported a 50% increase of activity on PGP^14^.

According to our data, C3-R201 and C2-H163 residues, which are close to each other in the catalytic pocket (Fig. 1D), were not major amino acids for C_55_-PP hydrolysis (25 and 27% relative activity, respectively), while they were essential for PGP hydrolysis (0.3 and 0.09% relative activity, respectively) (Table 3). Thus, the C2-His residue may not fulfill the assigned catalytic role in PgpB such as hypothesized, raising the question on which residue accomplishes this proton donor/acceptor in an acid/base process. In PgpB, the side chain carboxylate from E154 residue points towards the imidazole ring of H207, 5.6 Å apart, suggesting that this residue could fulfill the proton donor/acceptor function. To test this hypothesis, we then generated the E154A and the double E154A/H163A variants. The E154A variant displayed 60% of relative activity on both substrates and the double mutant displayed 10% and 0.03% of relative activity on C_55_-PP and PGP, respectively (Table 3). These results suggested that the second mutation has an additive effect over the first mutation. In the case H163 and E154 residues were to fulfill the same function in an alternative way, one would expect a synergistic effect of the double mutation. Therefore, the alternative E154 does not fit as likely.

An explanation would be that C_55_-PP hydrolysis in fact does not require a proton donor as the product can readily be released in its dianionic C_55_-PO_4_^2−^ form. In support to this, the acid dissociation constants of the phosphates of single chain polyprenyl phosphates in bilayer were reported to be 2.9 and 7.8 for the α and β phosphates, respectively^26^. In contrast, the release of PG from PGP absolutely requires the transfer of a proton to the oxygen at the junction between the glycerol and terminal phosphate. This proton would still be abstracted from H163 as suggested by the low activity of the corresponding mutant towards PGP.

### H207A variant shows high thermal stability upon phosphate binding

**Figure 4:**
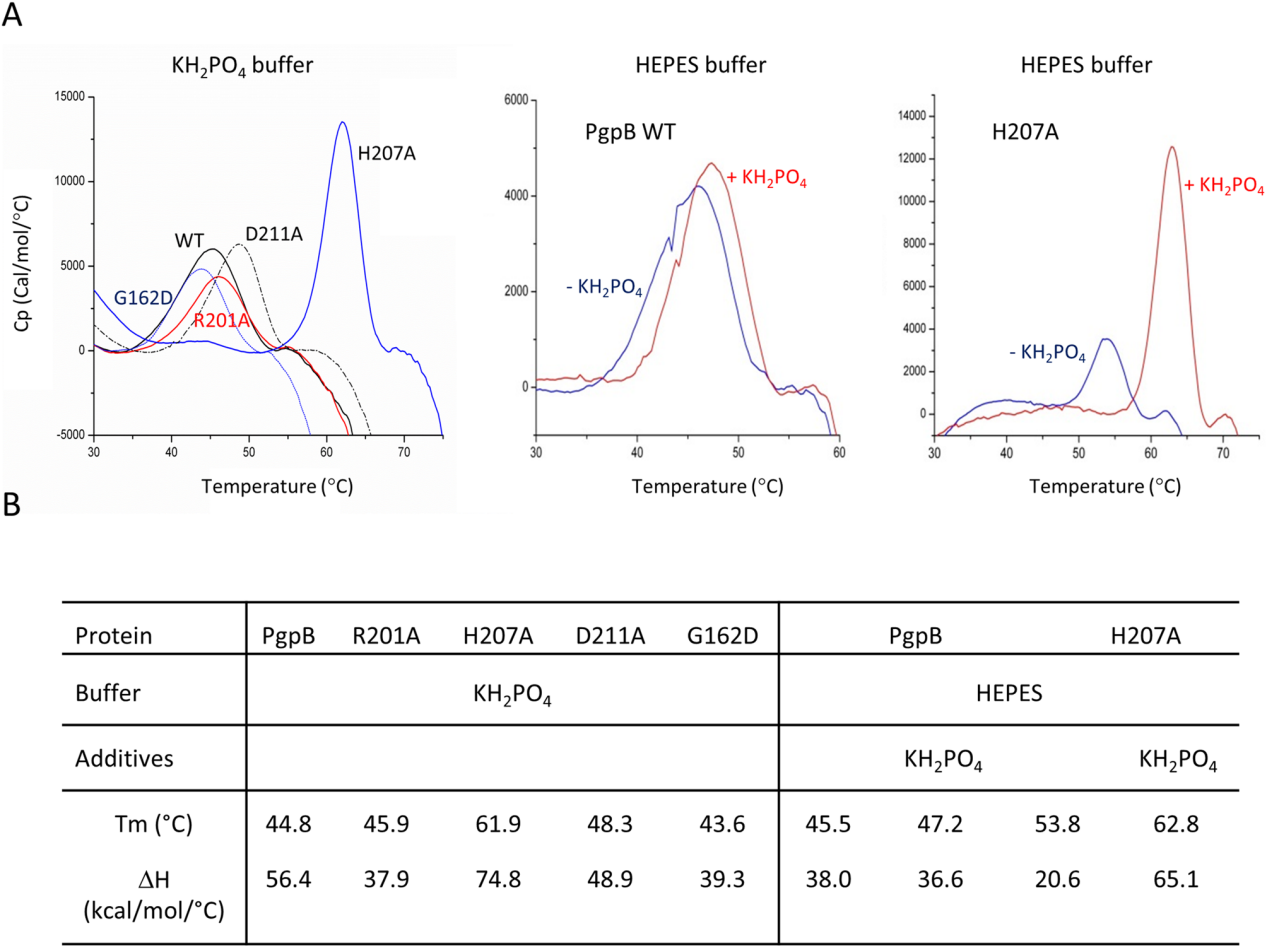
Thermal stability studies of PgpB variants. (**A**) DSC measurements were performed with PgpB variants at a protein concentration of 0.5 mg/ml in 20 mM potassium phosphate buffer, pH 6.0, or HEPES buffer supplemented or not with 20 mM KH_2_PO_4_. The fitting data are shown by lines in the graphs. (**B**) Estimated values of the specific enthalpy change (ΔH) and the peak of excess heat capacity (Tm).

The thermal stability of WT PgpB and variants was compared by differential scanning calorimetry (DSC). Interestingly, the H207A mutant showed a large increase of Tm (+ 17.1 °C) and denaturation enthalpy change (ΔH, + 18.4 kcal/mol/°C) as compared to WT (Fig. 4). These results strongly suggested that the H207A mutation has triggered a large structural change. These measurements were performed in phosphate buffer that was used for protein purification, which led us hypothesize that the change might occur through a differential phosphate-binding event. After purification of the proteins in HEPES buffer, the WT protein yielded a similar Tm but a lower ΔH (Fig. 4), and the addition of 20 mM KH_2_PO_4_ did not change these values. The H207A variant displayed a signal closer to that observed with WT, but upon KH_2_PO_4_ addition, both Tm and ΔH values raised greatly (Fig. 4). A similar behavior of the mutant was observed in the presence of inorganic pyrophosphate (+ 14.4 °C of Tm and + 74 kcal/mol/°C of ΔH).

The active site of PAP2s has evolved to stabilize the phosphorylated form of H207 to optimally trigger the dephosphorylation. This ideal position of phosphate cannot be reached in the absence of a covalent bond with the histidine because of steric hindrance. When H207 is changed in alanine, the steric hindrance does not occur anymore and the phosphate can adopt its most favorable position, stabilized by the side chains of R104, S161 and R201. The latter interaction would induce the movement of the TM3 helix closer to the core helix bundle, which could trigger further stabilization. These data then support an induced fit mechanism for PgpB that would occur upon phospho-enzyme intermediate formation.

### A mutation at the cytoplasmic face of PgpB affects C_55_-PP hydrolysis

Whether PAP2 C_55_-PP phosphatases are also involved in de novo synthesis of C_55_-P remains elusive in the absence of an identified cytoplasm-oriented C_55_-PP phosphatase. If this would be the case, de novo synthesized C_55_-PP would be flipped to the outer side of the membrane to be dephosphorylated and the product would be flipped back to the inner side to be used as lipid carrier. We then hypothesized that the PAP2s may be involved in the flip-flop of the lipid.

To identify residues that would be involved in the translocation of the anionic lipid substrate across the membrane, we mutagenized or deleted positively charged residues from cytoplasmic turns, the N- and C-termini and TMs (Supplementary Table S1). Out of 13 constructs, only the R7A variant failed to complement BWTetra-Ts*bacA* strain (Supplementary Table S5). The purified R7A variant displayed close Tm and ΔH values as compared to WT (Tm of 42.3 °C versus 43.9, ΔH of 28.9 kcal/mol/°C versus 33.5, respectively) and 17% of relative C_55_-PP phosphatase activity (Supplementary Fig. S4). The R7A residue is located at the cytoplasmic side of PgpB, at the end of TM1 and at the opposite side with respect to the entrance cleft, which make difficult to figure out how this mutation could affect the enzymatic activity, when the overall stability of the protein was conserved.

This variant reminded the R201A variant behaviour, i.e. their efficacy on C_55_-P pathway in vivo was impaired as judged from default of complementation while their enzymatic activity was relatively moderately affected suggesting they may disturb the same path in PgpB cycle. The purified R7A/R201A variant displayed similar Tm and ΔH values as the single R7A variant and 5.5% of relative activity on C_55_-PP, showing a simple additive effect of the mutations on the activity of the enzyme (Supplementary Fig. S4). This non-epistatic behaviour rather indicated that R7A and R201A mutations did not alter the same process of the PgpB catalytic cycle.

## Discussion

In the present study, we showed that PgpB was the only one among PAP2s (i.e. PgpB, YbjG, LpxT ang YnbD) and other lipid phosphatases (i.e. BacA, PgpA and PgpC) from *E. coli*, being active on very distinct substrates and capable of supplying both C_55_-P and PG simultaneously. While the disruption of *pgpB* did not apparently perturb the envelope integrity, its knockout together with *ybjG* and/or *lpxT* did it, as examined by DOC sensitivity assays. These phenotypes were further shown to likely originate from a defect in LPS biosynthesis. Interestingly, *pgpB* forms an operon with two genes encoding LapA and LapB proteins, which are involved in controlling the biosynthesis of LPS to ensure the proper balance between LPS and phospholipids biosyntheses^27–29^. The *lap* genes are located downstream of *pgpB* and are also transcribed from own promoters, which suggests a functional link between PgpB and LapA/LapB system. Knockout of *lap* genes causes severe growth defects, from permeability defects to lethality. The phenotypes of PAP2 deletion mutants should not originate from a polar effect of *pgpB* knockout because (1) the resistance cassette inserted for gene disruption was systematically removed afterwards, (2) the single Δ*pgpB* mutant did not display any growth or LPS defects, (3) the disruption of LpxT and YbjG also resulted in changes in the LPS pattern even though it did not increase DOC susceptibility and (4) *lapA* and *lapB* genes were also expressed from their own promoters. As being involved in the synthesis of membrane lipids and peptidoglycan, PAP2s also contribute to the homeostasis of cell envelope, i.e. the balanced synthesis of LPS, glycerophospholipids and peptidoglycan. PAP2 enzymes partially share the same function in a redundant way, i.e. for C_55_-PP dephosphorylation, while ensuring specific additional roles, i.e. glycerophospholipids synthesis for PgpB and LPS modification for LpxT. Simple depletions do not cause observable defects likely due to the redundancy of their C_55_-PP phosphatase activity, while multiple inactivation is not as neutral anymore for cell envelope integrity. Our observations and the genetic link between PgpB and LapA–LapB system strongly suggest the existence of an interplay between PAP2 enzymes and this central regulatory system in order to maintain cell envelope homeostasis, what must now be further investigated. Another important link between the synthesis of LPS and peptidoglycan concerns their common metabolic precursor, UDP-*N*-acetylglucosamine (UDP-GlcNAc), whose biosynthesis constitutes an essential point for controlling for the balanced synthesis of these two envelope elements.

Our PgpB mutagenesis study supports the central catalytic role played by C3-His (H207) and C3-Asp (D211) residues for both C_55_-PP and PGP hydrolysis. Surprisingly, Tong et al. reported a relatively high activity, i.e. 50% of activity as compared to the WT protein, for the D211A variant with PGP, while the activity was much more reduced with other glycerophospholipid substrates. We have no rational explanation for this discrepancy considering that both studies were performed in much the same way. Tong et al. quantified the release of phosphate, while we directly quantified the release of PG, however, the output should be similar. The main difference resides in the fact that Tong et al. used C16:1 PGP while we used saturated C16:0 PGP; however, we do not consider that as an explanation for such different catalytic behaviors.

The catalytic cycle of PAP2s was hypothesized to involve a third catalytic residue, the His residue from motif C2. Nevertheless, the mutation of the corresponding amino acid, H163 in PgpB, affects in very different ways the hydrolysis of C_55_-PP and PGP. The same discrepancy occurred with the mutagenesis of R201, which is close to H163 in the catalytic pocket. Our data suggest that, in contrast to PGP dephosphorylation, C_55_-PP hydrolysis does not require protonation of the leaving product and that C_55_-P is readily released in its dianionic phosphate form. The R201 residue could ensure the proper orientation of H163 side chain or the adequate stabilization of the bound PGP substrate in the catalytic pocket to facilitate proton shuttling. These conditions would again not be necessary for C_55_-PP hydrolysis. In the second step, our results suggest that the activation of a water molecule by H163 is no more required. Phospho-histidine is acknowledged as significantly less stable as compared to serine-, threonine- and tyrosine-phosphate, therefore, it is not clear whether the natural dephosphorylation rate of H207 would be sufficient to sustain efficient turn over or if another residue contributes to this process. This reduced role of H163 for pyrophosphate hydrolysis is compatible with the reduced activity on two DGPP substrates (about 25% and 20% residual activity for DGPP 18:1 and DGPP 8:0, respectively) and lower values for mono-phosphate substrates reported so far^13,14^, but like for the D211A mutant, we did not find any explanation for the increased activity (140% residual activity) reported by Tong et al. with the H163A mutant on PGP.

In PgpB structure, the TM3 is loosely packed to the core helix bundle generating a V-shaped cleft hypothesized to be the substrate entrance^13^. The physiological relevance of this TM3 translation, which was not observed in YodM and soluble PAP2s, is questioned. Upon substrate binding, the protein may undergo a conformational change with TM3 moving toward the core protein. The H207A variant showed a large gain of stability upon phosphate binding, suggestive of a conformation change that supports a repacking of the 6-TM helices in a substrate binding-induced conformational change. This large flexibility of PgpB could provide a much larger repertoire of potentially catalytic conformations explaining its promiscuous trait concerning substrate specificity.

BacA represents the other type of C_55_-PP phosphatases, whose structure strongly suggested that the protein might display a C_55_-P flippase activity^7,10^. Such an activity is required to relocalize the C_55_-P product back to the inner side of the membrane to end recycling. If BacA catalyzes the C_55_-P flip, the question arises as to whether PAP2 proteins also exert this function since BacA and PAP2s, except LpxT, complement each other in vivo^4^. Contrary to BacA, the structure of PgpB did not provide clear evidence for such a function. Nevertheless, the movement of TM3 helix of PgpB that would occur upon substrate binding may act as a piston pushing the polar head group of the first leaving product, the C_55_-P, across the plasma membrane while its hydrophobic tail would tip inside the membrane. The elucidation of the C_55_-P flip across a membrane now constitutes a fundamental issue and an important challenge in the field of bacterial cell wall biogenesis.

## Methods

### Bacterial strains, plasmids and media

All strains used are listed in Table 1. Plasmids and primers are listed in Supplementary Tables S1 and S4 in the Supporting information. Bacteria were grown in 2YT broth supplemented, when required, with ampicillin, kanamycin or chloramphenicol at 100, 50 and 25 µg/ml, respectively. DOC was added at 20 mg/ml final concentration in liquid or solid 2YT broth. For susceptibility assays towards ampicillin, Triton X100 and SDS, 2YT-agar plates were overlaid with the appropriate strain. A bacterial suspension at 10^8^ CFU/ml was prepared in 5 ml of sterile water. The plate was flooded with this suspension for 1 min to allow the bacteria to sediment before removing the excess of water. Drops of 5 μl of the compounds at various concentrations were added at the surface of the agar and the plates were incubated at 37 °C.

### Strains construction

The BWTetra-Ts*bacA* strain was generated from the DMEG8 strain^4^ (Table 1), which was first transformed by pMAK*bacA* (Cam^R^) plasmid and then the Δ*pgpB*::Kan^R^ cassette from DMEG4 was transferred by transduction with phage P1. The BW*pgpB*-single strain was constructed from DMEG10 by P1 transduction of Δ*pgpC*::Kan^R^ cassette from JW2544 strain obtained from the Keio collection^30^. The BWΔ*ynbD*::Kan^R^ strain was generated by Datsenko and Wanner method with primers Inact1-*ynbD* and Inact2-*ynbD*^31^. The BWΔ*ybjG*Δ*pgpB* was generated from DMEG12 by excision of the resistance cassette with the plasmid pCP20 expressing the Flp recombinase^31^. The BWPAP2-less strain was then generated by the successive transfers of Δ*ynbD*::Kan^R^ and Δ*lpxT*::Cam^R^ cassettes from the BWΔ*ynbD*::Kan^R^ and DMEG3 strains, respectively, into the BWΔ*ybjG*Δ*pgpB* recipient strain. The BWΔ*bacA*, BWΔ*lpxT*, BWΔ*pgpB* and BWΔ*ybjG* strains were generated by cassette excision from strains DMEG1, DMEG3, DMEG4 and DMEG2, respectively. The strains BWΔ*lpxT*Δ*pgpB* and BWΔ*bacA*Δ*lpxT*Δ*pgpB* were also generated by P1 transduction, followed by excision of the resistance cassettes. The strains expressing only one C_55_-PP phosphatase and either PBP1A or PBP1B were then generated by transduction of Δ*mrcA*::Cam^R^ or Δ*mrcB*::Cam^R^ from strains BWΔ*mrcA*::Cam^R^ and BWΔ*mrcB*::Cam^R32^, respectively, into the corresponding recipient strains. All strains were systematically verified by PCR.

### Plasmids construction

The plasmids carrying the different phosphatase-encoding genes were generated by PCR amplification of the corresponding ORFs with primers listed in Supplementary Table S4, followed by insertion of the amplicons at different restriction sites of the p*Trc*His30 and p*Trc*His60 expression vectors^33^ (the restriction sites and the vectors used are indicated in the Supplementary Tables S1 and S4). Site-directed mutagenesis of *pgpB* was performed on p*Trc*H60*pgpB* plasmid by using the “QuikChange II XL Site-Directed Mutagenesis Kit” from Agilent with couples of primers listed in Supplementary Table S4. All plasmid sequences were checked by sequencing.

### Chemicals

C_5_-PP (isopentenyl pyrophosphate), C_15_-PP (farnesyl pyrophosphate) and CDP-diacylglycerol were purchased from Sigma. [^14^C]C_5_-PP and [^14^C]PGP were from PerkinElmer. The [^14^C]C_55_-PP substrate was prepared as previously described by successive condensations of [^14^C]C_5_-PP onto C_15_-PP catalyzed by the purified UppS enzyme^9^. The 16:0 [^14^C]PGP substrate (1,2-dipalmitoyl-*sn*-glycero-3-[phospho-*rac*-(1′-(3′-phospho)glycerol)]) was synthesized by using the purified PgsA enzyme mixed with CDP-diacylglycerol and [^14^C]glycerol-3′-phosphate as previously described^21^. The *n*-dodecyl-β-d-maltoside (DDM) detergent was purchased from Anatrace and nickel-nitrilotriacetate-agarose (Ni^2+^-NTA-agarose) was from Qiagen. The different enzymes used for molecular biology techniques were from New England Biolabs, and DNA purification kits were from Macherey–Nagel. Primer synthesis and DNA sequencing were performed by Eurofins Genomics. All other materials were reagent grade and obtained from commercial sources.

### Analysis of LPS

The LPS were prepared according to the protocol already described^34^. They were analyzed by 15% SDS–polyacrylamide gel electrophoresis and visualized by silver staining as described previously^35^.

### Functional complementation assays

Functional complementation assays were performed as previously described^36^. Briefly, the *E. coli* thermosensitive strains were transformed by plasmids carrying the phosphatase-encoding genes to be tested for complementation. Isolated transformants were subcultured at 30 °C in liquid 2YT medium with ampicillin up to A_600nm_ = 0.5. The culture was then diluted 10^5^ fold in 2YT medium, and 100-µl aliquots were plated on two ampicillin-containing 2YT-agar plates which were incubated at either 30 °C or 42 °C for 24 h. When required, IPTG was added in the medium to increase gene expression. The colony forming units (CFU) were counted on each plate and functional complementation of conditional strains was evaluated by the capacity of the transformants to grow at both temperatures.

### Quantitative RT-PCR analysis

Total RNA were extracted from bacteria grown to the middle of exponential phase (A_600nm_ = 0.5) using RNeasy Protect bacteria Mini Kit system (Qiagen) according to the manufacturer’s instructions. cDNA synthesis was performed from 2 µg of total RNA with random hexanucleotides as primers using the SuperScript IV First Strand Synthesis system for RT-PCR (Invitrogen). The quantitative PCR reactions were carried out using DyNAmo ColorFlash SYBR Green qPCR kit (Thermo Scientific) and were run in a StepOnePlus Real-Time PCR system (Applied Biosystems). The data were analyzed with StepOne software v2.3 using ΔΔCt method and normalized using the housekeeping genes *rrsA*, *gyrA* and *ffh* as reference genes.

### Membrane extracts preparation

Membrane extracts were prepared as previously described^4^ with minor modifications. Bacteria were grown in 200 ml 2YT medium at 37 °C. When the A_600nm_ reached 1.8, the cells were harvested and washed with 30 ml of cold 20 mM Tris–HCl buffer, pH 7.4, containing 0.2 M NaCl, 10 mM 2-mercaptoethanol and 10% glycerol. They were disrupted by sonication and the membranes were then pelleted by centrifugation at 4 °C for 20 min at 200,000 × *g*. Membranes solubilization was carried out for 2 h in the same buffer supplemented with 2% (w/v) DDM. The solubilized proteins were then recovered in the supernatant after centrifugation at 4 °C for 20 min at 200,000 × *g*. Protein concentration was determined by using the Sigma bicinchoninic acid (BCA) assay system.

### Purification of PgpB

The purification of PgpB was performed as previously described^6^ with few modifications. *E. coli* C43(DE3) cells transformed by the p*Trc*H60*pgpB* plasmid or its mutagenized variants were grown at 37 °C in 2YT medium (1 l) containing ampicillin. When the A_600nm_ reached 0.8, IPTG was added at a final concentration of 1 mM and growth was continued for 3.5 h. Cells were then harvested (4,000 × *g*, 10 min) and resuspended in 40 ml of 20 mM potassium phosphate buffer, pH 6 (or alternatively in HEPES buffer, pH 6 for DSC measurements), 1 mM MgCl_2_, 20 mM 2-mercaptoethanol, 0.5 M NaCl and 10% glycerol (buffer A). They were disrupted by three successive passages through a French press, and the membrane and soluble proteins were separated by ultra-centrifugation at 100,000 × *g* for 1 h. In each case, the resulting pellet was washed 3 times in 20 ml buffer A and membranes were solubilized by incubation in 20 ml buffer A supplemented with 2% (w/v) DDM for 2 h at 4 °C. The solution was centrifuged (100,000 × *g*, 1 h) and the supernatant was incubated with 2 ml Ni^2+^-NTA-agarose and 10 mM imidazole at 4 °C overnight. The polymer was washed successively with 20 volumes of 10 mM and 30 mM imidazole-containing buffer A supplemented with 0.2% DDM. Final elution was performed with buffer A supplemented with 400 mM imidazole and 0.2% DDM, yielding a pure fraction of PgpB. This fraction was concentrated up to 1.5 mg/ml by ultrafiltration on Amicon Ultra centrifugal filter devices (Millipore) and thoroughly dialyzed against 20 mM phosphate or HEPES buffer, pH 6, 150 mM NaCl and 0.02% DDM before being stored at − 20 °C. Protein concentration was determined with a NanoDrop 2000 spectrophotometer (Thermo Scientific) based on the theoretical molar extinction coefficient of 96,605 M^−1^ cm^−1^.

### Differential scanning calorimetry measurements

DSC measurements were performed on a MicroCal VP-DSC calorimeter (Malvern Panalytical). Protein samples were prepared in 20 mM potassium phosphate or HEPES buffer, pH 6.0, 0.2 M NaCl, 10 mM 2-mercaptoethanol, 10% glycerol and 0.02% DDM. A scan rate of 1 °C/min and a protein concentration of 0.5 mg/ml were fixed for all DSC experiments. All thermodynamic data are given per mole of protein. The excess molar heat capacity function was obtained after a baseline subtraction, assuming that the baseline is given by the linear temperature dependence of the native-state heat capacity^37^. Buffer–buffer scans were recorded under the same conditions and subtracted from sample endotherms. The denaturation temperature, Tm, corresponds to the maximum of the DSC peak,the total denaturation enthalpy, ΔH, is the integrated area under the DSC peak. The uncertainty was 0.2 °C on the temperature and within 10% on the enthalpy.

### Phosphatase assays

Standard phosphatase assays were performed in 20-μl reaction mixtures containing 20 mM Tris–HCl, pH 7.5, 10 mM 2-mercaptoethanol, 150 mM NaCl, 0.6% DDM, 50 µM [^14^C]C_55_-PP or [^14^C]PGP, and enzyme, as previously described^9^.

## Supplementary information

Supplementary Information.
