# Supplementary material for: Insight into the dual function of lipid phosphate phosphatase PgpB involved in two essential cell-envelope metabolic pathways in Escherichia coli
Source: Sci Rep. 2020 Aug 6;10:13209. doi: 10.1038/s41598-020-70047-5 (PMC7413402; doi:10.1038/s41598-020-70047-5)
Supplement: Supplementary file 1 — Supplementary Information. [file 41598_2020_70047_MOESM1_ESM.pdf]

SUPPLEMENTAL MATERIAL

Insight into the dual function of lipid phosphate phosphatase PgpB involved in two essential cell-envelope metabolic pathways in *Escherichia coli*

Xudong Tian[1*], Rodolphe Auger[1*], Guillaume Manat[1], Frédéric Kerff[2], Dominique Mengin-Lecreulx[1], and Thierry Touzé[1#]

[1] Université Paris-Saclay, CEA, CNRS, Institute for Integrative Biology of the Cell (I2BC), 91198, Gif-sur-Yvette, France
[2]Centre d'Ingénierie des Protéines, InBioS, Université de Liège, Liège, Belgium

*These authors contributed equally

[#]To whom correspondence should be adressed : Thierry Touzé, Université Paris-Saclay, CEA, CNRS, Institute for Integrative Biology of the Cell (I2BC), 91198, Gif-sur-Yvette, France
thierry.touze@i2bc.paris-saclay.fr

Table S1. Plasmids

pMAK*bacA*	Cam^R	[4]
p*Trc*His60	Amp^R	[33]
p*Trc*His30	Amp^R	[33]
p*Trc*Bac30	p*Trc*His30 derivative for overproduction of N-His$_6$-BacA	[3]
p*Trc*H60*ybjG*	p*Trc*His60 derivative for overproduction of C-His$_6$-YbjG	This study
p*Trc*H60*lpxT*	p*Trc*His60 derivative for overproduction of C-His$_6$-LpxT	[5]
p*Trc*H60*pgpB*	p*Trc*His60 derivative for overproduction of C-His$_6$-PgpB	[6]
p*Trc*H30*ynbD*	p*Trc*His30 derivative for overproduction of N-His$_6$-YnbD	This study
p*Trc*H60*pgpA*	p*Trc*His60 derivative for overproduction of C-His$_6$-PgpA	This study
p*Trc*H60*pgpC*	p*Trc*His60 derivative for overproduction of C-His$_6$-PgpC	This study
pKD4	PCR amplification of Kan^R cassette for gene deletion	[31]
pCP20	Resistance cassette removal by Flp recombinase expression	[31]
pKD46	Resistance cassette insertion by lambda red recombinase expression	[31]
pA727	p*Trc*H60*pgpB* variant with PgpB K97A mutation	This study
pA730	p*Trc*H60*pgpB* variant with PgpB ΔV100 mutation	This study
pA733	p*Trc*H60*pgpB* variant with PgpB R104A mutation	This study
pA737	p*Trc*H60*pgpB* variant with PgpB P105A mutation	This study
pA741	p*Trc*H60*pgpB* variant with PgpB A158S mutation	This study
pA745	p*Trc*H60*pgpB* variant with PgpB F159A mutation	This study
pA762	p*Trc*H60*pgpB* variant with PgpB P160A mutation	This study
pA766	p*Trc*H60*pgpB* variant with PgpB S161A mutation	This study
pA771	p*Trc*H60*pgpB* variant with PgpB G162A mutation	This study
pA749	p*Trc*H60*pgpB* variant with PgpB G162D mutation	This study
pA798	p*Trc*H60*pgpB* variant with PgpB H163A mutation	This study
pA802	p*Trc*H60*pgpB* variant with PgpB T164A mutation	This study
pB244	p*Trc*H60*pgpB* variant with PgpB H150A mutation	This study
pA807	p*Trc*H60*pgpB* variant with PgpB S200A mutation	This study
pA812	p*Trc*H60*pgpB* variant with PgpB R201A mutation	This study
pA817	p*Trc*H60*pgpB* variant with PgpB L202A mutation	This study
pA822	p*Trc*H60*pgpB* variant with PgpB G205A mutation	This study
pA828	p*Trc*H60*pgpB* variant with PgpB H207A mutation	This study
pA833	p*Trc*H60*pgpB* variant with PgpB W208A mutation	This study
pA838	p*Trc*H60*pgpB* variant with PgpB P209A mutation	This study
pA843	p*Trc*H60*pgpB* variant with PgpB D211A mutation	This study
pA847	p*Trc*H60*pgpB* variant with PgpB L212A mutation	This study
pA852	p*Trc*H60*pgpB* variant with PgpB A215G mutation	This study
pA857	p*Trc*H60*pgpB* variant with PgpB S219A mutation	This study
pB089	p*Trc*H60*pgpB* variant with PgpB R6A mutation	This study
pB093	p*Trc*H60*pgpB* variant with PgpB R7A mutation	This study

pB232	p*Trc*H60*pgpB* variant with PgpB H57A mutation	This study
pB115	p*Trc*H60*pgpB* variant with PgpB R69A mutation	This study
pB099	p*Trc*H60*pgpB* variant with PgpB R71A mutation	This study
pB103	p*Trc*H60*pgpB* variant with PgpB K73A mutation	This study
pB107	p*Trc*H60*pgpB* variant with PgpB R181A mutation	This study
pB111	p*Trc*H60*pgpB* variant with PgpB R182A mutation	This study
pB118	p*Trc*H60*pgpB* variant with PgpB R183 mutation	This study
pB121	p*Trc*H60*pgpB* variant with PgpB R181A-R182A mutations	This study
pB124	p*Trc*H60*pgpB* variant with PgpB R182A-R183A mutations	This study
pB127	p*Trc*H60*pgpB* variant with PgpB R232A mutation	This study
pB194	p*Trc*H60*pgpB* variant with PgpB Δ236-254 (ΔC^{ter}) deletion	This study
pB877	p*Trc*H60*pgpB* variant with PgpB E154A mutation	This study
pB881	p*Trc*H60*pgpB* variant with PgpB E154A–H163A mutations	This study

Table S2. Phosphatase activities in membranes of *E. coli* mutants.

	Relative activities (% of WT)	
Substrate	C_{55}-PP	PGP
BW25113	100 +/- 2	100 +/- 3
BWΔ*pgpB*	96 +/- 11	7 +/- 8
BW*pgpB*-single	7 +/- 3	73 +/- 20

The C_{55}-PP and PGP phosphatase activities of BW25113, BWΔ*pgpB* and BW*pgpB*-single strains were measured and normalized by the quantity of proteins in each membrane extracts. The relative activities as compared to the WT strain membrane extracts are indicated. Each value is the mean of three independent measurements.

Table S3. Quantitative RT-PCR analysis of *pgpB* transcripts.

Plasmid	IPTG	PgpB expression (ratio of chromosomal expression)
none	-	1
*pTrc*H60*pgpB*	-	33 +/- 6
	+	2003 +/- 362

BW25113 cells carrying either no plasmid or p*Trc*H60*pgpB* were grown in 2YT medium in the presence or not of 1 mM IPTG up to A_{600nm} = 0.5. The total RNA was extracted and quantitative RT-PCR analysis was performed using appropriate primers (Table S4) and reference genes for normalization.

Table S4. Primers

Gene inactivation		
Inact1-ynbD	ACGGTTTCGATGGCGGTGCGTCGTGATAACTGAACGTCGGAACGT	*ynbD* inactivation
Inact2-ynbD	TAGATATTCAGTCCACATCTCAATCCACTTACCTGTTTTCCCATA	
Gene cloning[a]		
*ybjG*NcoI	GCGCCCATGGTGGAAAATTTGAATCTCTCTCTATTC	Cloning of *ybjG* in p*Trc*His60
*ybjG*BglII	CTTTTTAGCAGAGATCTGTCACGCACCCAGCC	
*ynbD*BamHI	GCGCGGATCCCTACAAGGCGCTGGCTGGTTATTGTTGC	Cloning of *ynbD* in p*Trc*His30
*ynbD*HindIII	GCGCAAGCTTTTACCTGTTTTCCCATAATCTCAGC	
*pgpB*NcoI	GCGCCCATGGCCATGCGTTCGATTGCCAGACGTACC	Cloning of *pgpB* in p*Trc*His60
*pgpB*BglII	GCGCAGATCTACTTTCTTGTTCTCGTTGCGCTAT	
*pgpB*BglII-ΔC^{ter}	CGCGAGATCTTGGCCCACAAATTCGTTGCGC	Cloning of *pgpB* ΔC^{ter} in p*Trc*His60
*pgpA*NcoI	GCGCCCATGGTGACCATTTTGCCACGCCATAAAG	Cloning *pgpA* in p*Trc*H60
*pgpA*BglII	GCGCAGATCTCGACAGAATACCCAGCGGCCAGTG	
*pgpC*NcoI	GCGCCCATGGTGGCAACTCACGAGCGTCGTGTGGTG	Cloning of *pgpC* in p*Trc*His60
*pgpC*BglII	GCGCAGATCTTTCCAGTTGCTGGAGTTCACCGCG	
pgpB site-directed mutagenesis[b]		
K97A	GTTAAATCCTGGATCGCAGACAAAGTCCAGG	
ΔV100	CCTGGATCAAAGACAAACAGGAACCACGACC	
R104A	CAAAGTCCAGGAACCAGCACCTTTTGTTATCTGG	
P105A	GTCCAGGAACCACGAGCTTTTGTTATCTGGCTG	
E154A	CACACTGGCAGAAAGCGACGGGGTTTGCCTTTC	
A158S	GAAAGAGACGGGGTTTAGCTTTCCTTCCGGTC	
F159A	GAGACGGGGTTTGCCGCTCCTTCCGGTCACAC	
P106A	CGGGGTTTGCCTTTGCTTCCGGTCACACG	
S161A	GGGTTTGCCTTTCCTGCCGGTCACACGATG	
G162A	GTTTGCCTTTCCTTCCGCTCACACGATGTTTG	
G162D	GTTTGCCTTTCCTTCCGATCACACGATGTTTG	
H163A	GCCTTTCCTTCCGGTGCCACGATGTTTGCTGC	
T164A	CTTTCCTTCCGGTCACGCGATGTTTGCTGCC	
S200A	CGGGAGTCATGGGAGCCCGCCTGCTGCTCGG	
R201A	GGAGTCATGGGAAGCGCCCTGCTGCTCGGG	
L202A	GTCATGGGAAGCCGCGCGCTGCTCGGGATGC	
G205A	GCCGCCTGCTGCTCGCGATGCATTGGCCACGC	
H207A	CTGCTGCTCGGGATGGCTTGGCCACGCGATCTG	
W208A	CTGCTCGGGATGCATGCGCCACGCGATCTGG	
P209A	CTCGGGATGCATTGGGCACGCGATCTGGTAG	
D211A	GCATTGGCCACGCGCTCTGGTAGTAGCTACG	
L212A	CATTGGCCACGCGATGCGGTAGTAGCTACGTTG	
A215G	GCGATCTGGTAGTAGGTACGTTGATTTCGTG	

S219A	GTAGCTACGTTGATTGCGTGGGCGCTGGTGG
R6A	GCGTTCGATTGCCGCACGTACCGCAGTGG
R7A	GTTCGATTGCCAGAGCTACCGCAGTGGGAG
H57A	GGCGTCATTACAGCTTTGATTTTATTCGG
R69A	GTTTCTCTGGTGTCTGGCTTTTCGCATTAAGG
R71A	GGTGTCTGCGTTTTGCCATTAAGGCTGCC
K73A	CTGCGTTTTCGCATTGCGGCTGCCTTTGTAT
R181A	GTTTGCTGTGGCCGGCTCGGCGAACGTTAAC
R182A	GCTGTGGCCGCGTGCGCGAACGTTAACC
R183A	GTGGCCGCGTCGGGCAACGTTAACCATTG
R181-182A	GGTTTGCTGTGGCCGGCTGCGCGAACGTTAACCAT
R182-183A	GCTGTGGCCGCGTGCGGCAACGTTAACCATTGC
R232A	CGTGGCTTGCGCAAGCAATTTGTGGGCC
Quantitative PCR	
198-pgpB-F	GTGTCTGCGTTTTCGCATTA
320-pgpB-R	ACAAAAGGTCGTGGTTCCTG
394-gyrA-F	TGAACGGTTCTTCCGGTATC
635-gyrA-R	ATGTGTTCCATCAGCCCTTC
1163-ffh-F	GCGCTAAGCCAGAAATCATC
1304-ffh-R	ATTCCGCCCTTCTTCATTTT
284-rrsA-F	CAGCCACACTGGAACTGAGA
400-rrsA-R	CCGAAGGCCTTTTTCATACA

[a]The restriction site used for the insertion of the amplicon in the expression vector is indicated in the primer's name and it is underlined in the sequence. [b]Of the two primers used for site-directed mutagenesis, only one sequence is indicated, the other sequence being its reverse complement.

Table S5. Functional complementation of thermosensitive strains by PgpB variants

Plasmid	protein	BWTetra-Ts*bacA*		BWPGPTs	
		- IPTG	+ IPTG	- IPTG	+ IPTG
p*Trc*His60	none	−	−	−	−
p*Trc*H60*pgpB*	PgpB	+	+	+	+
**N-terminus**					
pB089	R6A	+	+	+	+
pB093	R7A	−	+	+	+
**TM 2**					
pB232	H57A	+	+	+	+
**Cytoplasmic loop 1**					
pB115	R69A	+	+	+	+
pB099	R71A	+	+	+	+
pB103	K73A	+	+	+	+
**Cytoplasmic loop 2**					
pB107	R181A	+	+	+	+
pB111	R182A	+	+	+	+
pB118	R183	+	+	+	+
pB121	R181A-R182A	+	+	+	+
pB124	R182A-R183A	+	+	+	+
**C-terminus**					
pB127	R232A	+	+	+	+
pB194	Δ236-254(ΔC_{ter})	+	+	+	+

The plasmids carrying a copy of *pgpB* (wild-type or variants) were tested for their ability to restore the growth at 42°C of thermosensitive strains BWTetra-Ts*bacA* and BWPGPTs. +, normal growth at 42°C; - no growth at 42°C.

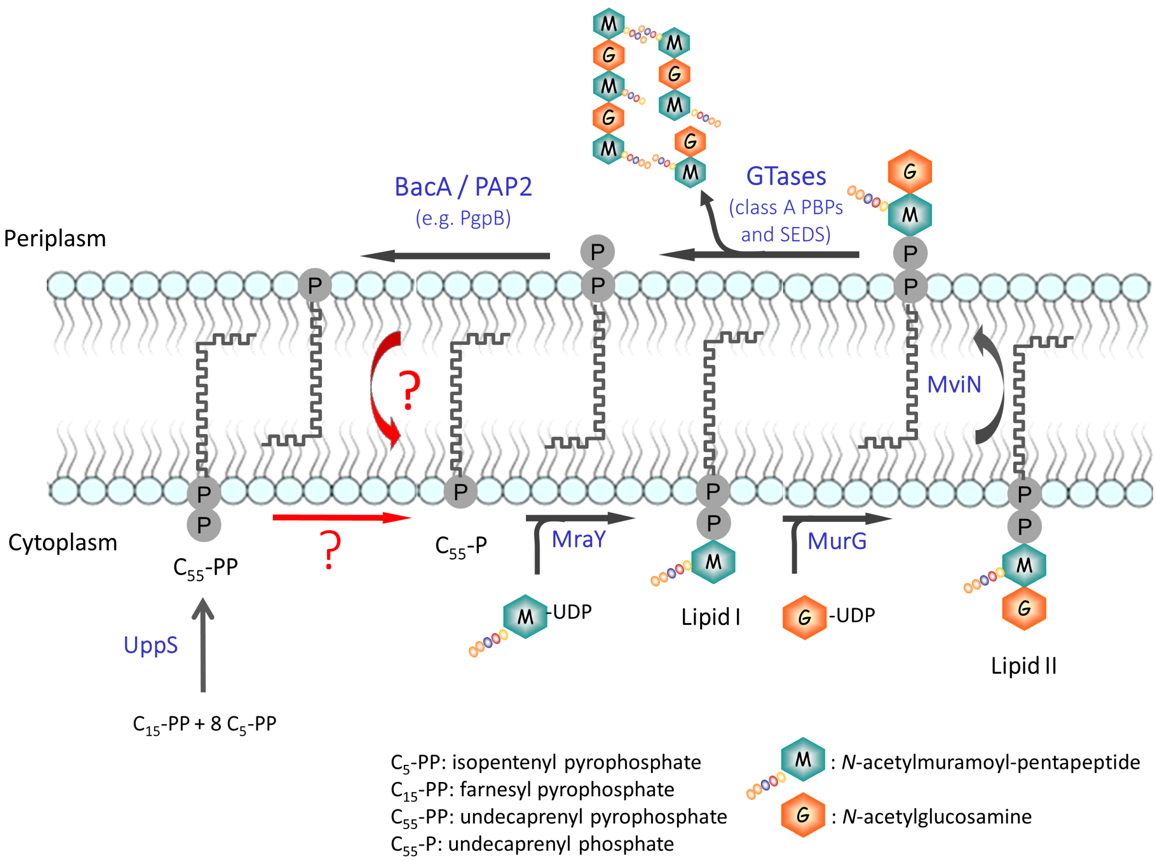

Figure S1. Schematic representation of C_{55}-P metabolism and peptidoglycan biosynthesis. The C_{55}-PP is *de novo* synthesized by UppS at the cytosolic face of the membrane; thereafter it is dephosphorylated, by a yet unknown enzyme, to yield the C_{55}-P lipid carrier. The MraY and MurG enzymes catalyze the successive transfers of phospho-*N*-acetylmuramoyl-pentapeptide and *N*-acetylglucosamine moieties from nucleotide precursors to C_{55}-P, yielding lipid II. The lipid II is flipped to the periplasmic face of the membrane, where glycosyltransferases (GTases) transfer the glycan-peptide moiety to the nascent polymer. GTases release C_{55}-PP as a by-product, which is then recycled through a dephosphorylation step by BacA or PAP2 enzymes and a flip back to the inner side, by a yet unknown mechanism, to enter a new cycle of peptidoglycan biosynthesis.

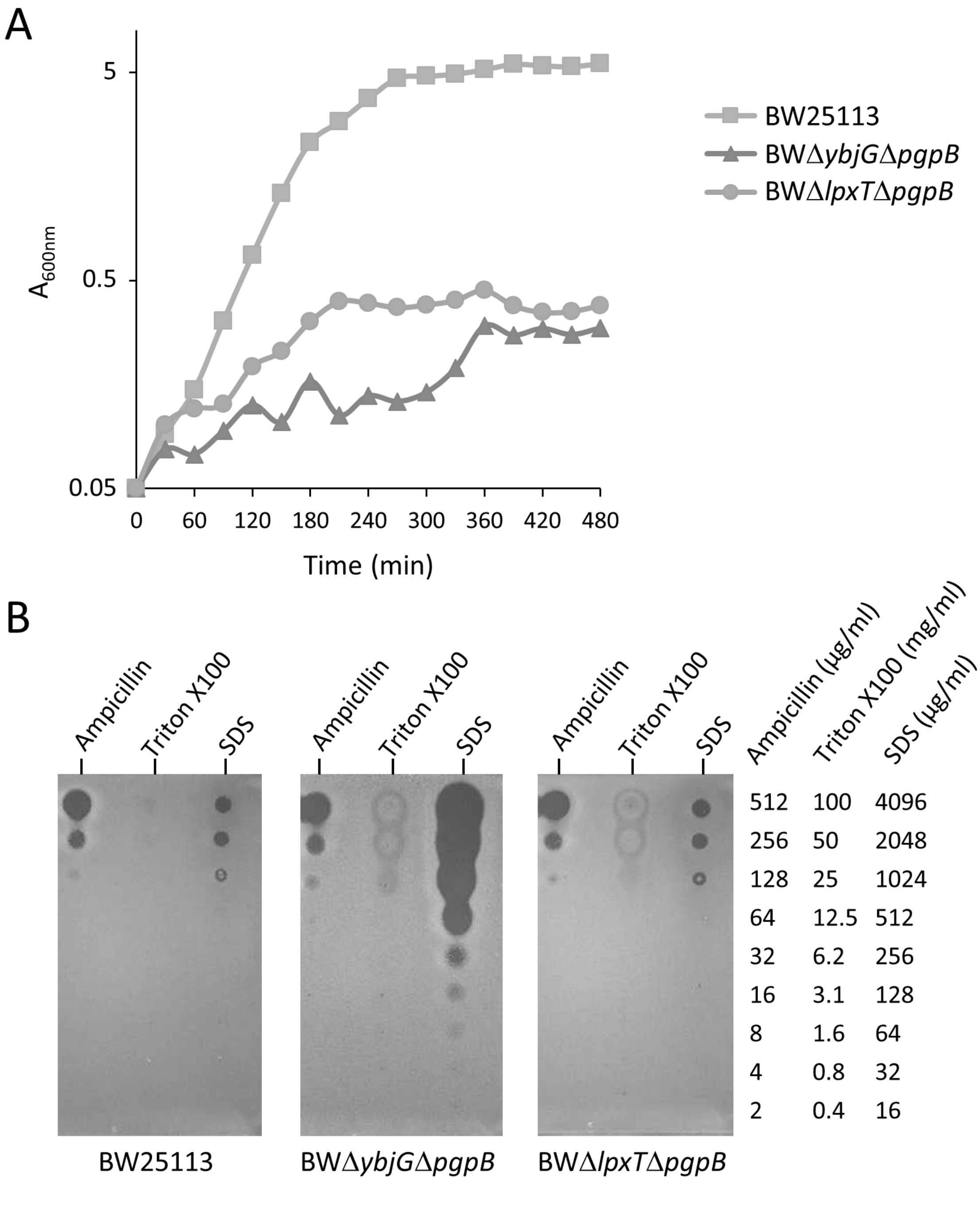

Figure S2. Multiple inactivation of PAP2s differentially sensitizes the cells to small hydrophobic compounds. (A) Cell growth was monitored at 37°C in 2YT medium containing 20 mg/ml of DOC. (B) 2YT-agar plates were overlaid with the indicated strains and 5 µl of serial dilutions (as indicated on the left) of ampicillin, Triton X100 or SDS were dropped on these plates.

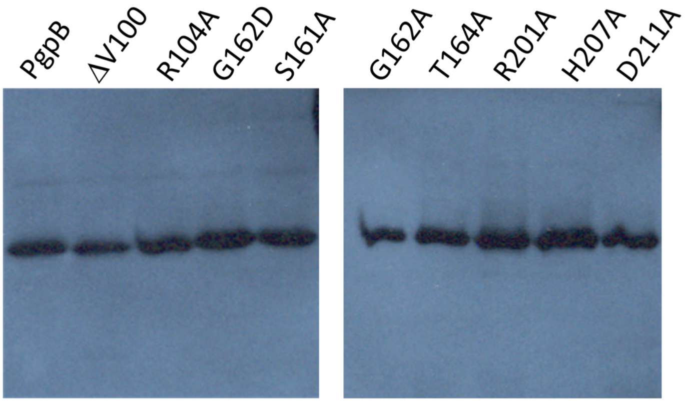

Figure S3. Expression of PgpB WT and variants presenting a default of complementation. Immunoblotting analysis of membrane fractions isolated from BW25113 cells producing PgpB and its variants from the p*Trc*His60 vector. Total DDM-solubilized membrane proteins (5 µg) were separated by SDS-PAGE and analyzed by immunoblotting with anti-His antibodies.

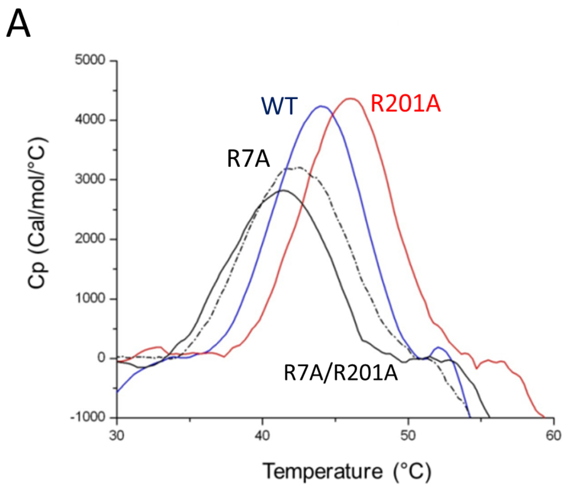

B

PgpB	C_{55}-PP phosphatase activity (% of WT)
WT	100
R201A	25 +/- 6.4
R7A	17 +/- 3.1
R201A/R7A	5.5 +/- 0.4

Figure S4. Thermal stability and activity of PgpB variants. (A) DSC measurements were performed with proteins at a concentration of 0.5 mg/ml in potassium phosphate buffer, pH 6.0. (B) The C_{55}-PP phosphatase activity of the variants is expressed as a percentage of the WT PgpB activity.
